# Buffalo Academy of Medicine

**Published:** 1900-10

**Authors:** Thomas F. Dwyer


### BUFFALO ACADEMY OF MEDICINE.

Reported by THOMAS F. DWYER, M. D., Secretary.

THE regular quarterly meeting of the Buffalo Academy of Medicine was held at the Academy rooms, Public Library Building, Tuesday, September 11, 1900. The president, Dr. MARCEL HARTWIG, called the meeting to order at 8.50 p.m.

The minutes of the last quarterly meeting were read and approved.

The council recommended the election of Drs. William C. Fritz, Julius Pohlman, Ray H. Johnson, Nelson W. Wilson, Charles A. Brownell, Isaac C. Munson, Frank W. Field, William L. Phillips, Herman B. Singer, Jacob S. Otto, and John W. Cameron, for resident membership, and Dr. E. W. Ewell, Lancaster, N.Y., for non-resident membership. They were separately balloted for and elected Fellows of the Academy.

The secretary read a report from the Council, to whom was referred the revision of the constitution and by-laws, giving the proposed changes which will be acted on at the next quarterly meeting. The section of medicine provided the following program: Clinical quantative estimation of proteid digestion in the stomach with demonstrations, Dr. A. L. Benedict.

Attendance, 21. Adjourned at 10.30 p.m.

---

### *Section on Surgery, September 4, 1900.*

Reported by FREDERICK H. MILLENER, M. D., Secretary.

The first regular meeting of this section for the season of 1900 and 1901, was held on September 4, 1900, twenty-eight Fellows being present.

Dr. MARSHALL CLINTON in the chair. The minutes of the previous meeting were read and approved.

The essayists of the evening were Drs. Vertner Kenerson and Herman Mynter.

Dr. VERTNER KENERSON read a paper entitled, Lymphangioma, in which he reported two cases, both occurring in infants said to have appeared shortly after birth; in one case the left side of the face was involved, and in the other the right shoulder. In both cases the operation was performed with fatal results from shock; the hemorrhage in both cases was comparatively small.

In the second case elaborate preparations were made for supplying the child with normal salt solution to prevent shock and to supply a substitute for the lymph which was lost in the previous case. The tumors were dingy blue in color, firm, not fluctuating, firmly adherent, no pulsation, no points of tenderness.

The affliction in these cases is great, the children are really monstrosities, the tumors increase with age and the individuals are not able to secure ordinary employment. Microscopic examinations showed that the skin and lymph spaces were involved and connected with deep lymphatics of the trunk; drawings and photographs were presented. The condition was suggested as being caused by congenital or acquired occlusion of the efferent trunk of a set of lymphatics which supply a particular part. He believes these cases to be operative, if done early and with proper precautions.

DISCUSSION.

Dr. Eugene Smith thought that fatality in the second case might be due to a possible toxic effect of salt solution, as very little blood had been lost and therefore no solution required.

Dr. Herman Mynter described a case of traumatic origin.

Dr. Chauncey P. Smith also thought the salt solution in this case toxic and described parallel cases.

Dr. Herman Mynter presented several very interesting cases of joint resection and described the pathology of lesions causing the same.

Meeting adjourned at 10.05.

